# Genomic analysis of metabolic pathway gene expression in mice

**DOI:** 10.1186/gb-2005-6-7-r59

**Published:** 2005-07-01

**Authors:** Anatole Ghazalpour, Sudheer Doss, Sonal S Sheth, Leslie A Ingram-Drake, Eric E Schadt, Aldons J Lusis, Thomas A Drake

**Affiliations:** 1 Department of Human Genetics, Department of Medicine and Department of Microbiology, Immunology and Molecular Genetics, and Molecular Biology Institute, University of California, Los Angeles, CA 90095-1679, USA; 2 Department of Pathology and Laboratory Medicine, University of California, Los Angeles, CA 90095-1732, USA; 3 Rosetta Inpharmatics LLC, Kirkland, WA 98034, USA

## Abstract

**Background:**

A segregating population of (C57BL/6J × DBA/2J)F2 intercross mice was studied for obesity-related traits and for global gene expression in liver. Quantitative trait locus analyses were applied to the subcutaneous fat-mass trait and all gene-expression data. These data were then used to identify gene sets that are differentially perturbed in lean and obese mice.

**Results:**

We integrated global gene-expression data with phenotypic and genetic segregation analyses to evaluate metabolic pathways associated with obesity. Using two approaches we identified 13 metabolic pathways whose genes are coordinately regulated in association with obesity. Four genomic regions on chromosomes 3, 6, 16, and 19 were found to control the coordinated expression of these pathways. Using criteria that included trait correlation, differential gene expression, and linkage to genomic regions, we identified novel genes potentially associated with the identified pathways.

**Conclusion:**

This study demonstrates that genetic and gene-expression data can be integrated to identify pathways associated with clinical traits and their underlying genetic determinants.

## Abstract 2

In order to evaluate metabolic pathways associated with obesity, global gene-expression data were integrated with phenotypic and genetic segregation analyses, identifying 13 metabolic pathways the genes of which are coordinately regulated in association with obesity. Four genomic regions were found to control the coordinated expression of these pathways and novel genes potentially associated with the identified pathways were identified.

## Background

Comprehensive high-throughput analytical techniques such as expression microarrays have brought about a rethinking of the possibility of understanding biology and disease at a global ('systems biology') level [1,2]. These techniques have been applied widely to the study of the time course of events in specific cells or organisms, and to different conditions for a given cell type or organism. More recently, there has been an appreciation of the possibility of using naturally occurring genetic variation as a means of generating perturbations, with the advantage of allowing identification of the individual genetic factors affecting the trait of interest in the segregating population [3]. We and others have begun to apply this approach to various model organisms where tracking of genetic segregation is feasible [4-10].

Traditional quantitative trait locus (QTL) analyses of complex traits in model organisms have focused on the identification of specific causative genes that differ between the originating strains and that are directly responsible for the variation in trait expression [11]. The availability of genome-wide expression data (or proteomic, metabolomic, or other such global analyses) to complement the measurements of the physiologic trait opens new opportunities for identifying specific biologic processes and genes that are involved in trait expression. As well as providing a means of evaluating many of the potential candidate genes responsible for a specific QTL, such data allow the identification of pathways and genes that have a role in the expression of the phenotype, either as intermediate players between the causative gene and the phenotype, or as being responsive to the trait [12].

We have been interested in using these approaches in mice to understand the pathogenesis of obesity and vascular disease, among other related conditions [6,13]. Metabolic dysregulation has been recognized to be an important element in the pathogenesis of these poorly understood conditions. An important dataset for these purposes is the (C57BL/6 × DBA/2)F2 (BxD) intercross presented in Schadt *et al*. [6]. This set comprises the microarray data from the liver of BxD F2 female mice fed an atherogenic diet for 4 months beginning at 12 months of age. In this study, we integrate the global gene-expression data with phenotypic and genetic segregation analyses to evaluate metabolic pathways associated with obesity in the BxD set. We show that this approach allows identification of specific pathways whose gene expression is coordinately regulated in association with obesity, defined genomic regulatory loci controlling the expression of these genes, and novel genes that are functionally associated with the identified pathways.

## Results

### Identification of gene sets/pathways associated with the subcutaneous fat pad mass trait

**Figure 1:**
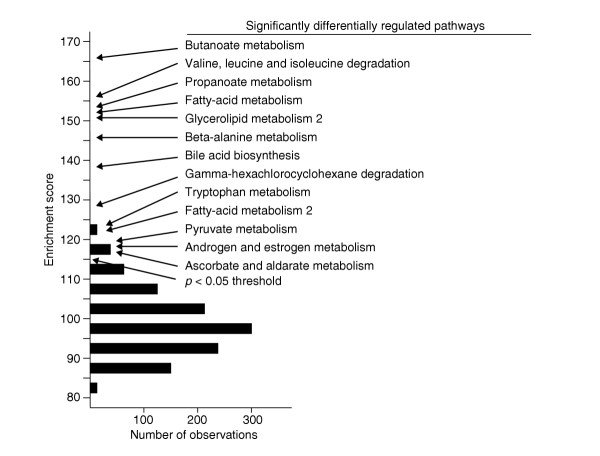
Significantly differentially regulated pathways identified by GSEA analysis. The histogram represents the frequency distribution of maximum ES scores obtained with GSEA analysis after 1,000 random permutations of class assignment (obese or lean). The location of the gray arrow corresponds to *p *= 0.05 (ES = 114.0), and pathways with scores greater than this (indicated by the black arrows) are listed.

To identify functionally related gene sets that are differentially perturbed in lean and obese mice of the BXD cross, two methods were applied: gene set enrichment analysis (GSEA) [14] and over-representation analysis using Fisher's exact test. For both of these, we began by selecting a set of 4,670 genes that were differentially expressed in the liver, filtered as described in the Materials and methods section. In both analyses, we used the same 378 gene sets compiled mainly from the Kyoto Encyclopedia of Genes and Genomes (KEGG) [15] and Biocarta [16] sources. GSEA was implemented as previously described [14]. To apply GSEA, the 4,670 filtered genes were ranked on the basis of significance of differential expression between the obese and lean groups of mice (identified as those mice in the upper and lower 15th percentiles for the subcutaneous fat-pad mass trait, respectively). The Kolmogorov-Smirnov (KS) test was then applied as described in [14] to test the null hypothesis that members of a given gene set are randomly ranked. This procedure generates an enrichment score (ES) for each gene set. The significance of the ES statistic was determined empirically by performing the analysis after random permutation of the grouping assignment of the mice, with the probability of falsely rejecting the null hypothesis determined by repeating the permutation procedure 1,000 times. This established that ESs greater than 114 allowed rejection of the null hypothesis at a level of 0.05. Using this empirically determined threshold for the ES statistic, we identified 13 gene sets that were differentially regulated between mice that have high and low subcutaneous fat-pad mass (Figure 1).

As a second approach, we used an analysis of over-representation of gene set members by applying the Fisher's exact test, as implemented by the Expression Analysis Systematic Explorer (EASE) software [17]. The results are shown in Table 1 and are comparable to those of the GSEA analysis. Ten of the 13 pathways identified as significantly differentially expressed using the GSEA approach were also identified as significantly differentially expressed by the EASE analysis after application of the Hochberg adjustment for multiple comparisons. Two of the other three gene sets identified by GSEA were the 12th- and 13th-ranked sets by the EASE analysis. One gene set (tetrachloroethene degradation) was identified as significant by the Fisher's exact analysis but not by GSEA.

### Relationships among pathway sets, gene expression, and the fat-mass phenotype

**Figure 2:**
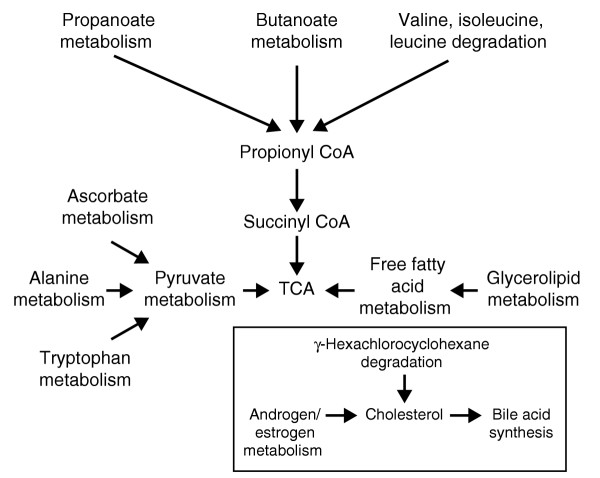
Relationship of the 13 differentially regulated pathways identified by GSEA. Nine pathways were related via the tricarboxylic acid (TCA) cycle and three other pathways are linked through cholesterol metabolism. These pathways contain a total of 170 genes, some of which are present in multiple pathways.

The gene sets identified as being differentially regulated between obese and lean mice represented metabolic pathways involved in energy and lipid metabolism. Note that genes can contribute to multiple pathways, as different pathways can be biologically interrelated. The majority of pathways feed into the tricarboxylic acid (TCA) cycle (Figure 2). The aggregate gene set from all 13 fat-mass-associated pathways comprises 170 genes, 150 of which were represented on the array. An examination of the correlation between relative gene-expression levels and the fat-mass trait showed that 68 of the 150 genes (45%) in the aggregate set were correlated at *p *< 0.05 (not corrected for multiple comparisons). A large majority of these genes (61/68) showed a significant positive correlation (that is, they were upregulated in obese compared with lean mice), with relatively few being negatively correlated (10%). Within individual pathway sets, between 42% and 63% of the genes were significantly correlated. The majority of correlated genes in a given set were always positively correlated. The magnitude of the correlations was comparable across the 13 pathways.

### Identification of genetic loci controlling differentially regulated pathway genes

We hypothesized that the composite set of pathways represented a functionally interrelated gene set responding to common genetic controls (referred to subsequently as the obesity-associated pathway set). Two approaches were taken to test for such common genetic controls. The first was to test for the enrichment of pathway gene-expression quantitative trait loci (eQTLs) along genomic intervals, and the second was principal component analysis (PCA) of the gene set, followed by QTL mapping of the derived principal components.

When eQTLs for the 4,670 most differentially expressed genes were mapped we found that there were 15,262 eQTLs with log_10 _of the odds (LOD) score greater than 2.0. Of these eQTLs, 278 corresponded to 77 genes represented in the 13 differentially regulated pathways identified above by the GSEA procedure. To examine whether there was an enrichment of pathway gene eQTLs among the eQTLs of the 4,670 most differentially expressed genes along the genomic intervals, we first divided the genome into sequential overlapping 20 centimorgan (cM) intervals, beginning a new interval every 10 cM. We then counted how many of the eQTLs overall for the 4,670 differentially expressed genes and how many of the eQTLs specific for the 77 pathway genes defined above mapped to each interval. In general, assuming under the null hypothesis that the genomic location of genes influencing the transcript levels of other genes in common pathways is independent, the expected number of pathway gene eQTLs mapping to each interval is (M/B) × K where B is 4,670, M is the subset from the 4,670 genes that have eQTLs mapping to the interval, and K is 77. To compute the statistics we applied Fisher's exact test. The *p*-values obtained for the statistics are shown in Table 2. For these calculations we applied a total of 180 tests overall, corresponding to 180 bins that comprise the entire genome. The significance levels of these tests are reported in Table 2 under 'adjusted *p*-value', which reflects the *p*-values after applying the Bonferroni correction criteria (multiplying the *p*-values by 180, the number of tests).

**Figure 3:**
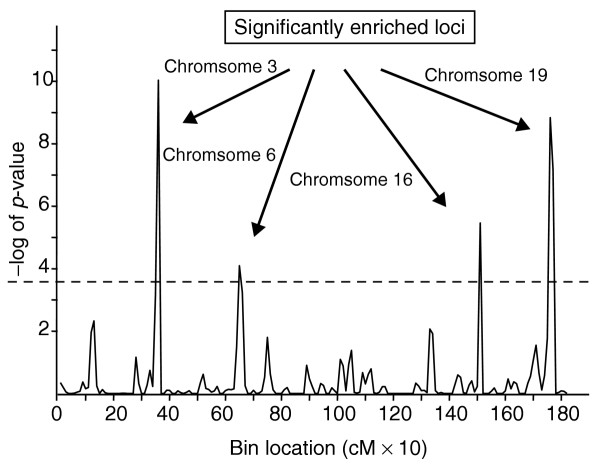
Identification of genomic regions controlling obesity-associated pathway gene expression. The *x*-axis represents sequential 10-cM intervals across the genome and the *y*-axis represents the negative log_10 _of adjusted Fisher's exact test *p*-value for enrichment of eQTLs in overlapping 20-cM bins. The dashed line corresponds to the *p*-value 0.05 after correcting for multiple comparison. The four significantly enriched regions on chromosomes 3, 6, 16, and 19 are indicated by arrows.

After correcting for multiple comparisons, significant over-representation of pathway gene eQTLs was obtained for four loci located on chromosomes 3 (80-100 cM), 6 (30-50 cM), 16 (1-20 cM), and 19 (20-50 cM) (Figure 3, Table 2). All pathways were represented at each locus and there were no apparent subgroups associated with some loci and not others. We also found that about half of the genes had eQTLs at two or more of the four loci.

An alternative approach that has been proposed to test for coordinated regulation of the genes in individual pathways is PCA [7,18]. PCA was applied using the gene sets from all 13 significant pathways identified by GSEA, and the aggregate set of 150 genes comprising these 13 pathways. The mean log ratios of the gene expression for all genes within these pathways across the F2 mice were subject to the analysis. Principal components (PC) that explained more than 5% of the variance of transcript levels of the genes within each pathway were then mapped as quantitative traits. Only three components showed significant LOD scores (> 4.3). These included the third PC for fatty acid metabolism-1 pathway (LOD 21.4), the fourth PC for γ-hexachlorocyclohexane degradation (LOD 14.8), and the fourth PC of the tryptophan degradation pathway (LOD 8.1). All these linkages mapped to the same locus at 36 cM on chromosome 19. The percentage of variance explained by each of these PCs was 8%, 8%, and 7%, respectively. QTL analyses of individual genes in the fatty-acid metabolism and γ-hexachlorocyclohexane degradation pathway revealed the presence of two *cis*-eQTLs at the chromosome 19 locus with very significant LOD scores: *Cyp2c40 *(LOD = 20.7) and *Cyp2c39 *(LOD = 12.4). To see whether these two genes were the major contributors to mapping of the PCs to chromosome 19, we carried out the analysis without these two genes for each of the pathways separately. The resulting components showed no evidence of significant linkage genome-wide.

### Relationship of pathway regulatory regions to the subcutaneous fat-mass trait

**Figure 4:**
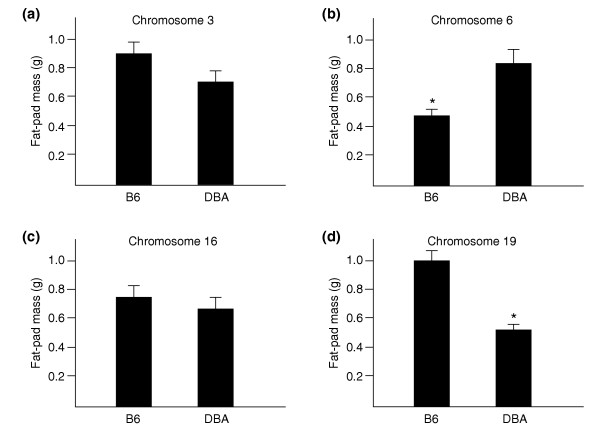
Homozygous allele effects on fat-pad mass of mice at the four loci identified to regulate obesity associated pathway gene members. **(a) **Chromosome 3 locus (peak marker at D3Mit86); **(b) **chromosome 6 locus (peak marker at D6Mit149); **(c) **chromosome 16 locus (peak marker at D16Mit100), and **(d) **chromosome 19 locus (peak marker at D19Mit63). B6, C57BL/6J; DBA, DBA/2J. *p-value < 0.05 calculated using two-sample *t*-test.

The loci identified above are genomic regions with apparent regulatory control of pathway genes that are differentially perturbed between obese and lean mice. The locus allele effects on the subcutaneous fat-mass trait, using the nearest markers at each locus, are shown in Figure 4. The loci on chromosomes 6 and 19, which involve the greatest number of pathway genes, showed the strongest effects and in fact had been identified as being linked to adiposity in previous studies [6,19]. Consistent allele effects were observed among the subset of genes from the aggregate pathway set that had eQTLs at both the chromosome 6 and chromosome 19 loci (that is, a gene that showed a positive correlation with fat mass showed greater expression with the B6 versus DBA homozygous mice at the chromosome 19 locus, and the reverse at the chromosome 6 locus).

### Use of expression data for prioritization of candidate gene selection at the chromosome 19 locus

The chromosome 19 locus had the greatest impact on the expression of pathway set gene expression, and it was of interest to identify the candidate responsible genes for further study. For candidate genes regulated at the level of expression (that is, the gene controls its own transcript abundance), an eQTL will be present proximal to the physical location of the gene; we have referred to such an eQTL as a *cis*-eQTL [6]. *Cis*-eQTLs were defined as any gene with eQTLs mapping to 20 Mb (approximately 10 cM) on either side of the gene's physical location. This definition reflects the relative accuracy of the 13 cM dense BxD QTL map. The decision to choose such a definition for *cis*-eQTLs is supported by the work of Doss *et al*. [20], who showed that this definition is reasonable by validating the *cis *nature of such defined eQTLs and by showing the extreme bias in the mapping of these *cis *eQTLs to regions which are not identical by descent between the B6 and DBA strains of mice.

We identified genes with significant *cis*-acting eQTL (LOD score > 4.3) that map to chromosome 19 between 20 and 50 cM, and determined the genomic location of each gene using the University of California Santa Cruz (UCSC) Genome Browser (build 33, mm5, May 2004). Of 249 eQTLs mapping to the chromosome 19 locus, 19 overlapped the physical location of their respective gene (Table 3), thus establishing them as primary candidate genes. Of these, eight were significantly correlated with the fat-mass trait, making them particularly attractive candidates.

### Integration of trait, expression, and mapping data to identify novel genes potentially related to the obesity-associated gene set

The above analyses were restricted to the genes that had been assigned to gene sets in the starting databases such as KEGG. To identify novel genes that may belong to the same aggregate group of obesity-associated pathway genes, we filtered the 4,670 differentially regulated genes first for correlation with the fat-mass trait at *p *< 0.05 (uncorrected, corresponding to a correlation coefficient of approximately 0.25) and subsequently for the presence of eQTLs falling within the identified regulatory regions for both chromosome 6 (30 to 50 cM interval) and 19 (20 to 50 cM interval). A total of 117 genes were identified that were not members of one of the 13 obesity-associated pathways. These include 20 genes with defined relationships to the starting pathways (based on published literature), but also 28 expressed sequence tags (ESTs) with no functional annotation. A complete list is provided in Additional data file 1.

## Discussion

This study shows that the incorporation of genome-wide gene-expression data with traditional phenotypic trait data in a mouse intercross population enables the identification of regulated metabolic pathways and the genomic regions that control the expression of the constituent pathway genes. In contrast to studies with genetically altered mice, we have studied a heterogeneous population where the genetic variations leading to the phenotypes and the changes in gene expression are all naturally occurring. In this work, we identified metabolic pathway gene sets with altered gene expression in association with the subcutaneous fat-pad mass trait, but the concept is applicable to other phenotypic traits where the target organ examined would be expected to show changes in relevant pathway gene expression. While gene-expression changes are not sufficient to imply actual metabolite flux through a pathway [21], changes in gene expression are likely to reflect pathway involvement in a given condition.

Two approaches were used to identify gene sets whose transcripts were altered in association with the fat-mass trait: the GSEA method and Fisher's exact test. These yielded comparable results, although the GSEA appeared to be more sensitive. Central to both is the availability or construction of predefined gene sets to test. We used two primary sources, the KEGG database, composed primarily of traditional metabolic pathways, and the Biocarta database, composed of gene sets of varying type. The total number of genes represented in these was relatively low (just under 2,000). There are certain to be biologically important gene sets as yet unrecognized, as well as currently uncategorized genes that belong in the existing sets. Our approach allows tentative functional assignment to such uncategorized genes when they share properties of correlation of expression levels with the phenotypic trait and co-localization of eQTL with the trait QTL.

Consistent with an obesity trait, the gene sets identified were of pathways directly or indirectly related to energy metabolism. Within any given pathway, only about half of the genes showed a correlation between transcript levels and fat mass, consistent with the concept that regulation of a metabolic pathway requires the control of only selected elements rather than all constituents. It is of interest that the pathways are at least loosely interconnected, and several of the genes examined have been shown to influence adipose tissue mass in other studies. For example, of the total of 150 pathway genes represented on the microarrays, the gene with the highest differential expression between obese and lean mice was amine oxidase copper-containing 3 (*Aoc3*) (12.5-fold change in expression level). This observation is consistent with the study in which overexpression of human *AOC3 *in mice resulted in an increase in weight and subcutaneous white adipose tissue when the animals were fed an atherogenic diet for 15 weeks [22]. Ten of the 13 pathways are connected through the generation of products involved in the TCA cycle (Figure 2). The other three pathways (bile acid biosynthesis, androgen/estrogen metabolism, and γ-hexachlorocyclohexane degradation) are involved in cholesterol metabolism. Coordinated control of cholesterol and fatty-acid synthesis has been recognized for some time [23]. The production of estrogen and androgen requires cholesterol as a precursor and the activity of the cytochrome P450 family, members of which were significantly upregulated in the obese F2 mice. In the bile acid pathway, cholesterol serves as a substrate that is converted to bile acids through the activation of 7-α-hydroxylase, the transcript for which is also significantly elevated in obese mice. Lastly, studies show that γ-hexachlorocyclohexane administration has been shown to increase serum cholesterol and very low-density lipoprotein (VLDL) levels [24]. Several genes occur multiple times in these 13 pathways (for example, the aldehyde dehydrogenase gene family (*Aldh1a1*, *Aldh2*, and *Aldh3a2*)). These enzymes are involved in the generation of acetate, which can be converted to acetyl CoA. The family is also involved in ascorbate/aldarate metabolism, valine, leucine, and isoleucine degradation, propanoate metabolism, fatty-acid metabolism, glycerolipid metabolism, β-alanine metabolism, bile acid biosynthesis, and pyruvate metabolism pathways (eight out of the 13 pathways). The increased expression of the aldehyde dehydrogenases such as *Aldh3a2 *in mice with higher subcutaneous fat-pad mass is consistent with recent studies that correlate hyperinsulinemia with increased hepatic expression of *Aldh3a2 *[25].

We were able to identify specific genomic regions on chromosomes 3, 6, 16, and 19 that regulated these pathways. Gene transcript levels are themselves quantitative traits for which genetic control regions can be identified by QTL analysis (eQTLs), just as for a traditional physiologic trait [4-6,26]. Loci that coordinately regulate pathway genes were identified by analyzing for over-representation of identified pathway-associated gene eQTLs among eQTLs of the set of most differentially expressed genes in a given genomic region. A possible criticism of this approach is the use of eQTLs with low LOD scores. While a given eQTL with a low LOD score cannot be considered significant in the genome-wide context on its own, the co-localization of multiple related eQTLs with LOD scores that may not meet the genome-wide significance criteria is highly unlikely to have occurred by chance, as we show in Table 2. It is unlikely that such eQTL clustering results from correlated traits linking to a particular marker by chance, given that the percent variation explained by any such marker is modest (typically less than 10%) and given that the correlation between traits is also modest (typically explaining less than 30% of the variation between the traits). Therefore, because the covariance between the two traits is small, it is unlikely that the covariance between the two traits captures the by-chance covariance between the traits and marker. The other approach that we used to identify pathway regulatory loci - PCA - proved less useful. When used on random or less correlated groups of genes, PCA spreads the variance components of the dataset over several different component vectors, thereby diluting the very signal one is trying to capture. Other techniques used for the decomposition of multivariate data such as non-negative matrix factorization [27] are being explored and may offer a more effective approach for this type of analysis.

The loci on chromosomes 6 and 19 had the greatest number of eQTLs, and were the two loci identified in previous studies to be significantly linked with the subcutaneous fat-mass trait. It is of interest that the most significant locus controlling this fat-mass trait is located on distal chromosome 2 [19], which was not represented among the loci we identified here. In the analysis reported in Schadt *et al*. [6], the loci on chromosomes 2 and 19 were associated with distinct subsets of mice, identified by differing expression profiles of the most differentially regulated genes. The gene sets we identified were associated with the chromosome 19 locus (and others) but not the chromosome 2 locus. There are several possible explanations (not mutually exclusive). One is that the responsible 'pathways' or gene sets involved with the chromosome 2 locus are not represented in our sets. A second is that too few genes of an associated pathway are differentially regulated to detect over-representation. A third is that the associated pathways primarily involve tissues that were not arrayed. That is, because only liver expression data were available, expression differences in other relevant tissues such as adipose, muscle, brain, or gut could be primarily related to the chromosome 2 QTL, but were not detected as these tissues were not examined.

We show that there are hotspots of eQTL activity for pathway gene members along the genome. One explanation for the presence of these hotspots is the presence of a major regulator gene within each locus that coordinately regulates obesity-related pathways. Alternatively, these loci may contain several such regulators that are closely linked. It is well known that clusters of duplicated genes with related functions occur commonly in the genome. Other clusters with diverse genes but related functions, such as the *H-2 *locus, are also known. Yet another possible explanation is that the genes with eQTL mapping to a common hotspot are in linkage disequilibrium (LD). Two or more genes that differ genetically between the two strains and that are in LD will show correlated expression levels [20]. This spurious correlation could lead to a more convoluted *trans*-eQTL hotspot for these genes which is more difficult to interpret [20]. We determined whether this situation might be occurring in our data and saw that in the 77 active pathway genes only 13 have *cis*-eQTLs for which there is a minimal clustering of physical location (data not shown). On this basis we conclude that LD is unlikely to explain the hotspots identified in this study.

An approach to prioritizing candidate genes using eQTL information is presented for the chromosome 19 locus. This uses the concept that for genes regulated at the level of transcription, *cis*-acting regulation can be identified by the coincidence of an eQTL for a gene and its physical location. We have experimentally verified that the majority of such putative *cis*-eQTLs are real [20]. This approach relies, however, on variations affecting transcript levels and will miss candidate genes with variations affecting protein function or post-transcriptional modification, as well as variants associated with alternative splicing. In addition, the list of candidate genes is limited to genes represented on the microarray chip.

## Conclusion

In conclusion, this study presents one approach for using gene-expression microarray data in conjunction with genetic segregation to identify metabolic pathways that are altered in association with obesity, and to identify specific genomic regions that exert regulatory control over these pathways. These findings contribute, along with other approaches developing genetic regulatory networks [12], to the ultimate goal of understanding disease pathophysiology at the systems biology level.

## Materials and methods

### Animals, tissue collection, and gene-expression profiling

This study, along with others previously reported [6,19,28], was based on data originating from an F2 intercross carried out between mouse strains C57BL/6J and DBA/2J. This intercross encompassed a two-phase study as described in Colinayo *et al*. [28], using 142 female F2 mice with genome-wide genotyping initially, followed by an additional 144 female mice to confirm specific QTLs (with genotyping limited to selected loci). Subsequently, genome-wide expression microarray analyses were obtained on portions of stored liver samples; 111 of these were from the initial 142 mouse set that was reported by Schadt *et al*. [6], and an additional 44 were from a second set that lacked genome-wide genotype data.

All F2 progeny were fed a chow diet until 12 months of age and then put on a high-fat high-cholesterol atherogenic diet containing 0.5% cholic acid for 4 months (diet 90221; Harlan Teklad). At 16 months of age these mice were euthanized and liver and other organs were removed and frozen at -70°C for subsequent RNA and DNA extraction. Body weight was determined before organ removal and adipose tissue depots were removed and weighed for a subset of 96 animals in the phase 1 group.

RNA extraction and expression microarray profiling were carried out as described using a custom ink-jet microarray (Agilent Technologies) containing 23,574 60mer oligonucleotide non-control probes for profiling gene expression in liver of the F2 mice [6]. Other tissues were not available for analysis. The microarray data discussed in this publication have been deposited in the Gene Expression Omnibus [29] of the National Center for Biotechnology Information (NCBI) and are accessible through GEO Series accession number [GSE:2008]. The genotype and the phenotype of F2 mice are given in Additional data file 2. Overall, 155 F2 mice had liver array data, 111 had both array and genome-wide genotype information, and 69 had array, genotype, and fat-mass trait data available.

### Linkage analysis

QTL analysis was performed using mean log_10 _expression ratio values (averaged over fluor-reverse pairs) as quantitative traits for the 111 mice that were part of the phase 1 group described above. The linkage map was constructed using microsatellite markers at an average density of 13 cM using MapManager QTX version 0.30 and QTL Cartographer version 2.0 [30-32]. For each trait, the LOD score was calculated at 1 cM intervals using standard interval mapping.

### Gene set and pathway assembly

A total of 378 gene sets were developed for gene set enrichment analysis (GSEA). Gene sets (118) representing metabolic pathways were obtained from KEGG [15,33,34]. Ten gene sets comprising genes that are highly correlated across 46 tissues in mice were selected [35]. These data were obtained using the SOURCE database [36,37]. Seven pathways were manually curated from the published literature for genes involved in insulin signaling. Two hundred and forty-four gene sets were obtained by querying all the pathways available at Biocarta [16]. The latter set comprises both signaling and metabolic pathways submitted to the Biocarta server by independent scientists. Gene sets composed of more than 50 members were divided into smaller sets to avoid bias related to gene set size in the GSEA analysis [38].

### Microarray data filtering and ranking

A subset of genes represented on the microarray were selected on the basis of differential expression across all the 155 mice arrayed, irrespective of phenotype. This was done to ensure a starting gene set that was active and differentially expressed in liver among mice. To filter for these genes, the expression ratio and the corresponding *p*-value for differential expression (compared to the control pool) for each gene were calculated as described [6,39]. Genes with *p*-values of 0.05 or less in 10 or more F2 mice were defined as differentially expressed, which yielded a set of 4,670 genes. To study gene subsets (within these 4,670 genes) that are associated with obesity, the mice comprising the upper and lower 15th percentiles of the subcutaneous fat-pad mass trait were selected. The average and standard deviation of the mean log ratio of expression of each differentially expressed gene for these two groups of mice were calculated and used for the GSEA and the Fisher's exact test analyses described below.

### Gene set enrichment analysis

Gene sets that are differentially regulated in relation to the subcutaneous fat-pad mass trait were identified using the GSEA method as described in [14]. The groups of mice analyzed were obese and lean F2 mice as defined above. To calculate the significance of the enrichment score (ES) assigned to each pathway, class labels of lean and obese mice were randomly permuted and ESs were recalculated 1,000 times. The cutoff for significance of ESs in the original analysis was defined as the score above the 50th highest score calculated in the permutation, corresponding to an empirical *p*-value of 0.05.

### Fisher's exact test analysis

The test subset was composed of transcripts identified as being the 20% most differentially regulated between obese and lean groups of mice. Fisher's exact test was applied as implemented in the Expression Analysis Systemic Explorer (EASE) program [17], downloaded from the Database for Annotation, Visualization, and Integrated Discovery (DAVID) [40,41]. The pathway set we compiled was incorporated as a file into the program for use in the analysis (LocusLink and pathway identifier for each gene associated with each pathway). Genes identified as being differentially regulated as described above, but not belonging to an identified pathway or gene set were included with a designation 'unclassified', so that the EASE program would perform the Fisher's exact analysis using the full set of differentially regulated genes, rather than just those associated with an identified pathway or gene set. The set of all analyzed transcripts on the array for which unique LocusLink identifiers were available was entered as the population set in the analysis, although in the EASE analysis this set is limited to the genes that are represented in the pathway being evaluated.

### *cis*-eQTL identification

We adopted the same *cis*-eQTL definition as described in [20]. An eQTL was defined as a proximal or *cis*-eQTL when the eQTL mapped within a 20 megabase (Mb) window on either side of the physical location of the gene. This corresponds to an approximately 20 cM segment of the genetic map, which is the approximate level of accuracy for a QTL given a genetic map with an average inter-marker distance of 13 cM. The following approach was taken to equate cM locations on the genetic map (constructed by the QTL software for this specific cross) with the Mb locations of the physical map, in order to be able to define eQTL as *cis *or *trans*. The physical location of the gene was obtained from the UCSC Genome Browser (build 33, mm5, May 2004). To map the genetic map location of the eQTL to the approximate equivalent location on the physical map, the physical map locations of the microsatellite markers immediately adjacent (proximal and distal) to the eQTL were identified from the UCSC genome browser (build 33, mm5, May 2004). Then the Mb 'location' of the eQTL was determined by interpolation based on the relative location of the eQTL between the markers on the genetic map.

### Principal components analysis

PCA was performed as implemented in the R statistical software version 1.8.1 [42] using the 'base' package. The principal components and eQTL were mapped using the standard interval mapping procedures implemented in MapManager QTX version 0.30 and composite interval mapping implemented in the Windows QTL Cartographer software version 2.0 [30-32].

## Additional data files

The following additional data are available with the online version of this paper. Additional data file 1 is a table listing genes that were not included in any tested gene set but are significantly correlated with subcutaneous fat mass and have eQTLs on chromosomes 6 and 19 (ordered alphabetically by gene name). Additional data file 2 is a table listing genotype and fat-mass trait data for the mice used in this study.

## Supplementary Material

Additional File 1 A table listing genes that were not included in any tested gene set but are significantly correlated with subcutaneous fat mass and have eQTLs on chromosomes 6 and 19 (ordered alphabetically by gene name). Click here for file

Additional File 2 A table listing genotype and fat-mass trait data for the mice used in this study. Click here for file

## Figures and Tables

**Table 1:** Top 13 differentially regulated pathways identified by EASE analysis ranked by their corresponding *p*-values

Gene category	*p*-value	Adjusted *p*-value
Fatty-acid metabolism	6.91 E-08	1.48 E-05
Tryptophan metabolism	3.72 E-06	2.94 E-04
Butanoate metabolism	4.12 E-06	2.94 E-04
Glycerolipid metabolism	7.45 E-06	3.99 E-04
Valine, leucine, isoleucine degradation	4.22 E-05	1.60 E-03
Gamma-hexachlorocyclohexane degradation	4.48 E-05	1.60 E-03
Beta-alanine metabolism	6.10 E-05	1.86 E-03
Bile acid biosynthesis	1.54 E-04	4.13 E-03
Ascorbate and aldarate metabolism	1.54 E-03	3.33 E-02
Tetrachloroethene degradation	1.56 E-03	3.33 E-02
Androgen and estrogen metabolism	1.87 E-03	3.64 E-02
Pyruvate metabolism	3.72 E-03	7.56 E-01
Propanoate metabolism	6.12 E-03	9.93 E-01

**Table 2:** Chromosomal loci with significant enrichment for obesity-associated pathway gene eQTLs relative to eQTLs of all 4,670 most differentially expressed genes

Chromosome (cM)	Total number of genes with eQTL	Expected number of pathway genes with eQTL	Observed number of pathway genes with eQTL	*p*-value	Adjusted *p*-value
3 (80-100)	25	1	9	9.24 E-11	1.68 E-08
6 (30-50)	836	14	28	8.05 E-05	1.47 E-02
16 (0-20)	431	7	21	3.44 E-06	6.27 E-04
19 (20-40)	891	15	38	1.45 E-09	2.63 E-07
19 (30-50)	709	12	31	6.77 E-08	1.23 E-05

**Table 3:** Nineteen candidate genes with *cis*-eQTL in chromosome 19 locus

*cis*-QTL gene symbol	Physical location of the gene (Mb)	Estimated physical location of the eQTL (Mb)	Correlation with subcutaneous fat mass
*RIKEN cDNA 9530025L08*	29.5	48.6	-0.29*
*Cyp2c40*^†^	39.1	40.6	0.24
*Col17a1*	47.0	40.6	-0.25*
*Scd2*	43.6	40.6	-0.19
*Slk*	46.9	41.8	-0.07
*Cyp2c37*	39.3	37.0	0.32*
*Cyp2c39*^†^	38.8	40.6	0.03
*Gsto1*	47.2	41.8	0.26*
*Rnf134*	46.4	48.6	0.05
*Cutc*	43.1	39.4	0.25*
*Cpn1*	43.3	39.4	0.28*
*Cnnm1*	42.8	46.2	0.27*
*Acsl5*	54.6	37.0	0.30*
*RIKEN cDNA 1810018P12*	32.0	35.7	0.24
*Pik3ap1*	40.6	39.4	-0.07
*RIKEN cDNA 0610010D20*	41.4	41.8	0.10
*RIKEN cDNA 4833409A17*	42.1	40.6	-0.21
*RIKEN cDNA 4930538D17*	45.7	38.2	-0.22
*RIKEN cDNA 5730455O13*	37.4	40.6	0.14

*Significant correlation of transcript levels with subcutaneous fat mass (*p *< 0.05). ^†^The two genes that solely contributed to the significant LOD score of the fatty-acid metabolism and γ-hexachlorocyclohexane degredation principal components to chromosome 19 (see text for details).
