# Supplementary material for: Genomic analysis of metabolic pathway gene expression in mice
Source: Genome Biol. 2005 Jul 1;6(7):R59. doi: 10.1186/gb-2005-6-7-r59 (PMC1175990; doi:10.1186/gb-2005-6-7-r59)
Supplement: Additional File 1 — A table listing genes that were not included in any tested gene set but are significantly correlated with subcutaneous fat mass and have eQTLs on chromosomes 6 and 19 (ordered alphabetically by gene name). [file gb-2005-6-7-r59-S1.doc]

**Supplementary Table 1**. Genes that are not associated with any pathway but are significantly correlated with subcutaneous fat mass and have eQTLs on chromosome 6 and 19. (ordered alphabetically by the “Gene Name”)

| Gene Name | Gene Symbol | **Correlation with subcutaneous fat mass** | **Go Category Associated with Energy and Lipid Metabolism** |
| --- | --- | --- | --- |
| 1-acylglycerol-3-phosphate O-acyltransferase 2 (lysophosphatidic acid acyltransferase, beta) | Agpat2 | 0.27 | phospholipid biosynthesis |
| 4-nitrophenylphosphatase domain and non-neuronal SNAP25-like protein homolog 1 (C. elegans) | Nipsnap1 | 0.35 |  |
| actin related protein 2/3 complex, subunit 1B | Arpc1b | -0.29 |  |
| adenine phosphoribosyl transferase | Aprt | -0.63 |  |
| adiponectin receptor 2 | Adipor2 | 0.31 | Lipid metabolism, fatty acid metabolism, fatty acid oxidation |
| agmatine ureohydrolase (agmatinase) | Agmat | 0.31 |  |
| AI875925 | EST | 0.44 |  |
| aldehyde dehydrogenase family 6, subfamily A1 | Aldh6a1 | 0.44 |  |
| apolipoprotein B editing complex 1 | Apobec1 | -0.27 | lipoprotein metabolism |
| AW494273 | EST | 0.33 |  |
| BCL2-associated athanogene 4 | Bag4 | 0.27 |  |
| carbohydrate kinase-like | Cark1 | 0.26 |  |
| carnitine acetyltransferase | Crat | 0.26 | acyl-CoA metabolism |
| cathelicidin antimicrobial peptide | Camp | -0.27 |  |
| cDNA sequence BC005662 | BC005662 | -0.26 |  |
| cDNA sequence BC028528 | BC028528 | -0.28 |  |
| CNDP dipeptidase 2 (metallopeptidase M20 family) | Cndp2 | -0.26 |  |
| complement component factor h-like 1 | Cfhl1 | 0.36 | Apolipoprotein, |
| core promoter element binding protein | Copeb | -0.33 |  |
| cysteine-rich motor neuron 1 | Crim1 | -0.35 |  |
| cytochrome c-1 | Cyc1 | 0.27 | electron transport;oxidative phosphorylation |
| cytochrome P450, family 4, subfamily a, polypeptide 14 | Cyp4a14 | 0.30 | electron transport |
| cytosolic acyl-CoA thioesterase 1 | Cte1 | 0.35 | acyl-CoA metabolism;long-chain fatty acid metabolism |
| dehydrogenase/reductase (SDR family) member 8 | Dhrs8 | 0.31 |  |
| deleted in polyposis 1-like 1 | Dp1l1 | 0.32 |  |
| dimethylglycine dehydrogenase precursor | Dmgdh | 0.25 |  |
| electron transferring flavoprotein, dehydrogenase | Etfdh | 0.41 | electron transport |
| endothelial cell growth factor 1 (platelet-derived) | Ecgf1 | 0.32 |  |
| enoyl coenzyme A hydratase 1, peroxisomal | Ech1 | 0.40 | fatty acid metabolism |
| Eph receptor B1 | Ephb1 | 0.38 |  |
| fatty acid desaturase 2 | Fads2 | 0.29 | stearoyl-CoA 9-desaturase activity, electron transport |
| Fc receptor, IgG, alpha chain transporter | Fcgrt | 0.45 |  |
| fructose bisphosphatase 1 | Fbp1 | 0.31 | gluconeogenesis |
| G protein-coupled receptor 35 | Gpr35 | -0.31 |  |
| G0/G1 switch gene 2 | G0s2 | 0.51 |  |
| glutathione S-transferase omega 1 | Gsto1 | 0.26 |  |
| glycoprotein (transmembrane) nmb | Gpnmb | -0.30 |  |
| glyoxalase 1 | Glo1 | 0.40 |  |
| glyoxylate reductase/hydroxypyruvate reductase | Grhpr | 0.43 |  |
| guanine nucleotide binding protein (G protein), gamma 2 subunit | Gng2 | -0.30 |  |
| guanine nucleotide binding protein (G protein), gamma transducing activity polypeptide 2 | Gngt2 | -0.27 |  |
| histone cell cycle regulation defective interacting protein 5 | Hirip5 | 0.40 |  |
| immunoglobulin superfamily, member 6 | Igsf6 | -0.27 |  |
| integrin beta 1 (fibronectin receptor beta) | Itgb1 | -0.29 |  |
| interferon consensus sequence binding protein 1 | Icsbp1 | -0.28 |  |
| interferon gamma receptor | Ifngr | -0.31 |  |
| IQ motif containing GTPase activating protein 1 | Iqgap1 | -0.31 |  |
| kallikrein B, plasma 1 | Klkb1 | 0.26 |  |
| KH domain containing, RNA binding, signal transduction associated 3 | Khdrbs3 | 0.47 |  |
| kynureninase (L-kynurenine hydrolase) | Kynu | 0.31 | amino acid metabolism |
| legumain | Lgmn | -0.37 |  |
| lipase, endothelial | Lipg | 0.38 | Lipid Metabolism |
| lysosomal-associated protein transmembrane 5 | Laptm5 | -0.30 |  |
| methionine sulfoxide reductase A | Msra | 0.37 |  |
| microsomal triglyceride transfer protein | Mttp | 0.27 | lipid transport;protein lipidation;triacylglycerol metabolism |
| myeloid-associated differentiation marker | Myadm | -0.31 |  |
| neutrophilic granule protein | Ngp | -0.28 |  |
| odd Oz/ten-m homolog 3 (Drosophila) | Odz3 | 0.44 |  |
| open reading frame 28 | ORF28 | 0.40 |  |
| ORM1-like 3 (S. cerevisiae) | Ormdl3 | 0.37 |  |
| pantothenate kinase 1 | Pank1 | 0.34 | Coenzyme A biosynthesis |
| peroxisomal biogenesis factor 11a | Pex11a | 0.39 |  |
| peroxisomal trans-2-enoyl-CoA reductase | Pecr | 0.29 | Fatty acid elongation |
| phenylalkylamine Ca2+ antagonist (emopamil) binding protein | Ebp | 0.34 | cholesterol biosynthesis, sterol biosynthesis |
| phosphatidylcholine transfer protein | Pctp | 0.40 | lipid binding, lipid transport |
| pleckstrin homology domain containing, family B (evectins) member 1 | Plekhb1 | 0.43 |  |
| progesterone receptor membrane component 2 | Pgrmc2 | 0.45 |  |
| proline rich membrane anchor 1 | Prima1 | 0.39 |  |
| protein tyrosine phosphatase, non-receptor type 18 | Ptpn18 | -0.27 |  |
| protein tyrosine phosphatase, non-receptor type substrate 1 | Ptpns1 | -0.28 |  |
| putative high mobility group box 2 pseudogene | LOC15352 | -0.32 |  |
| Ras association (RalGDS/AF-6) domain family 2 | Rassf2 | -0.29 |  |
| RAS p21 protein activator 3 | Rasa3 | -0.27 |  |
| Ras-related GTP binding D | Rragd | -0.26 |  |
| retinol dehydrogenase 6 | Rdh6 | 0.43 | retinol dehydrogenase activity, metabolism |
| ri|1300008M07|R000011O23||2154 | EST | 0.36 |  |
| ri|1700108L22|ZX00077B14||1706 | EST | 0.31 |  |
| ri|2010005F17|ZX00043H02||1460 | EST | 0.49 |  |
| ri|2510029O03|ZX00048A13||1650 | EST | -0.40 |  |
| ri|2610014E05|ZX00045A19||2337 | EST | 0.34 |  |
| ri|2810004E20|ZX00045P14||2015 | EST | 0.38 |  |
| ri|4933404M19|PX00019F10||1119 | EST | 0.59 |  |
| RIKEN cDNA 0610009E20 gene | 0610009E20Rik | 0.39 |  |
| RIKEN cDNA 0710001B24 gene | 0710001B24Rik | 0.38 |  |
| RIKEN cDNA 1300012D20 gene | 1300012D20Rik | 0.51 |  |
| RIKEN cDNA 1700020G04 gene | 1700020G04Rik | 0.50 |  |
| RIKEN cDNA 2010321J07 gene | 2010321J07Rik | 0.39 |  |
| RIKEN cDNA 2310057K14 gene | 2310057K14Rik | -0.35 |  |
| RIKEN cDNA 2400008B06 gene | 2400008B06Rik | 0.37 |  |
| RIKEN cDNA 2410021P16 gene | 2410021P16Rik | 0.37 | acyl-CoA dehydrogenase activity, electron transport |
| RIKEN cDNA 2410118P20 gene | 2410118P20Rik | -0.37 |  |
| RIKEN cDNA 2610034P21 gene | 2610034P21Rik | 0.65 |  |
| RIKEN cDNA 2810009O15 gene | 2810009O15Rik | 0.43 |  |
| RIKEN cDNA 2810457I06 gene | 2810457I06Rik | -0.32 |  |
| RIKEN cDNA 3000002F19 gene | 3000002F19Rik | 0.41 |  |
| RIKEN cDNA 4921501M20 gene | 4921501M20Rik | -0.26 |  |
| RIKEN cDNA 4930431B09 gene | 4930431B09Rik | -0.30 |  |
| RIKEN cDNA 4930474F22 gene | 4930474F22Rik | 0.42 |  |
| RIKEN cDNA 9030616G12 gene | 9030616G12Rik | 0.38 |  |
| RIKEN cDNA 9630055N22 gene | 9630055N22Rik | 0.40 |  |
| RIKEN cDNA D630032B01 gene | D630032B01Rik | 0.44 |  |
| SA rat hypertension-associated homolog | Sah | 0.30 | fatty acid biosynthesis, fatty-acid ligase activity |
| serine (or cysteine) proteinase inhibitor, clade B, member 1a | Serpinb1a | 0.55 | regulation of protein catabolism |
| serine hydroxymethyl transferase 1 (soluble) | Shmt1 | 0.29 |  |
| serum/glucocorticoid regulated kinase 2 | Sgk2 | 0.36 |  |
| sirtuin 5 (silent mating type information regulation 2 homolog) 5 (S. cerevisiae) | Sirt5 | 0.35 |  |
| solute carrier family 25 (mitochondrial carrier, dicarboxylate transporter), member 10 | Slc25a10 | 0.40 |  |
| solute carrier family 25 (mitochondrial carrier, Graves disease autoantigen), member 16 | Slc25a16 | 0.32 |  |
| solute carrier family 35 (UDP-N-acetylglucosamine (UDP-GlcNAc) transporter), member 3 | Slc35a3 | 0.48 |  |
| solute carrier family 38, member 1 | Slc38a1 | -0.28 |  |
| thioether S-methyltransferase | Temt | 0.34 |  |
| transforming growth factor, beta receptor II | Tgfbr2 | -0.26 |  |
| tumor suppressor candidate 4 | Tusc4 | 0.41 |  |
| uridine-cytidine kinase 1 | Uck1 | 0.68 |  |
| vanin 1 | Vnn1 | 0.53 |  |
| zinc finger protein, multitype 1 | Zfpm1 | 0.27 |  |
| zinc finger, DHHC domain containing 6 | Zdhhc6 | 0.29 |  |
